# Human airway and nasal organoids reveal escalating replicative fitness of SARS-CoV-2 emerging variants

**DOI:** 10.1073/pnas.2300376120

**Published:** 2023-04-17

**Authors:** Cun Li, Jingjing Huang, Yifei Yu, Zhixin Wan, Man Chun Chiu, Xiaojuan Liu, Shuxin Zhang, Jian-Piao Cai, Hin Chu, Gang Li, Jasper Fuk-Woo Chan, Kelvin Kai-Wang To, Zifeng Yang, Shibo Jiang, Kwok-yung Yuen, Hans Clevers, Jie Zhou

**Affiliations:** ^a^Department of Microbiology, School of Clinical Medicine, Li Ka Shing Faculty of Medicine, The University of Hong Kong, Hong Kong, China; ^b^State Key Laboratory of Emerging Infectious Diseases, The University of Hong Kong, Hong Kong, China; ^c^Centre for Virology, Vaccinology and Therapeutics, Hong Kong Science and Technology Park, Hong Kong, China; ^d^Department of Otolaryngology-Head and Neck Surgery, Precision Medicine Center, Nanfang Hospital, Southern Medical University, Guangzhou 510515, China; ^e^Carol Yu Centre for Infection, The University of Hong Kong, Hong Kong, China; ^f^State Key Laboratory of Respiratory Disease, National Clinical Research Center for Respiratory Disease, Guangzhou Institute of Respiratory Health, The First Affiliated Hospital of Guangzhou Medical University, Guangzhou 510163, China; ^g^Key Laboratory of Medical Molecular Virology, Institute of Infectious Disease and Biosecurity, School of Basic Medical Sciences, Fudan University, Shanghai 200032, China; ^h^Oncode Institute, Hubrecht Institute, Royal Netherlands Academy of Arts and Sciences, and University Medical Center Utrecht, 3584 CT Utrecht, the Netherlands; ^i^Pharma, Research and Early Development of F. Hoffmann-La Roche Ltd, CH-4070 Basel, Switzerland

**Keywords:** SARS-CoV-2, organoids, viral fitness, syncytium formation

## Abstract

The high transmissibility of SARS-CoV-2 Omicron subvariants was generally ascribed to immune escape. It remained unclear whether the emerging variants have gradually acquired replicative fitness in human respiratory epithelial cells. We sought to evaluate the replicative fitness of BA.5 and earlier variants in physiologically active respiratory organoids. BA.5 exhibited a dramatically increased replicative capacity and infectivity than B.1.1.529 and an ancestral strain wildtype (WT) in human nasal and airway organoids. BA.5 spike pseudovirus showed a significantly higher entry efficiency than that carrying WT or B.1.1.529 spike. Notably, we observed prominent syncytium formation in BA.5-infected nasal and airway organoids, albeit elusive in WT- and B.1.1.529-infected organoids. BA.5 spike-triggered syncytium formation was verified by lentiviral overexpression of spike in nasal organoids. Moreover, BA.5 replicated modestly in alveolar organoids, with a significantly lower titer than B.1.1.529 and WT. Collectively, the higher entry efficiency and fusogenic activity of BA.5 spike potentiated viral spread through syncytium formation in the human airway epithelium, leading to enhanced replicative fitness and immune evasion, whereas the attenuated replicative capacity of BA.5 in the alveolar organoids may account for its benign clinical manifestation.

## Significance

The high transmissibility of SARS-CoV-2 Omicron subvariants was largely ascribed to immune escape. We assessed the intrinsic fitness of BA.5 and the prior variants in adult stem cell-derived human respiratory organoids, which faithfully simulated the human upper and lower respiratory epithelium. We demonstrated that the higher entry efficiency and fusogenic activity of BA.5 spike enabled viral spread through syncytium formation in the human nasal and airway organoids, leading to dramatically enhanced replicative fitness, whereas BA.5 showed attenuated replication in human alveolar organoids. The results suggested that SARS-CoV-2 has evolved to gain increased replicative fitness and become well adapted in epithelial cells of human airways.

SARS-CoV-2 has evolved more rapidly since late 2021. The first Omicron variant B.1.1.529 was identified in Botswana and South Africa in November 2021 (1). Three subvariants BA.1, BA.2, and BA.3 were detected shortly thereafter (2). Subsequently, BA.2 outcompeted BA.1 and became the dominant variant globally. BA.4 and BA.5 were detected in South Africa in early 2022 (3), and shared an identical spike gene sequence. Relative to BA.2, BA.4 and BA.5 evolved additional spike mutations, including 69 to 70 deletion, L452R, and F486V. Since early August 2022, BA.5 outcompeted BA.2 and became the dominant variant worldwide.

Despite the occurrence of extrapulmonary complications, COVID-19 is primarily a respiratory infection. The human airway, from the nasal cavity to the terminal bronchiole, is lined with the airway epithelium, which consists of four major epithelial cell types, i.e., ciliated, goblet, basal, and club cells. Alveolar sacs, the functional units of gas exchange, are covered with the alveolar epithelium consisting of two cell populations, type I and type II alveolar epithelial cells. The airway epithelium, especially the epithelium covering the nasal–pharyngeal region, also known as the upper respiratory tract, is the primary infection site of SARS-CoV-2. Meanwhile, the infection may further disseminate to distal bronchioles and/or alveoli and cause lower respiratory infection or pneumonia. Human primary airway/bronchial epithelial cells might be the most biologically relevant tools to model SARS-CoV-2 infection (4). Yet, the limited expandability restricted their utility for routine experimentations. Respiratory organoids derived from inducible pluripotent stem cells (iPSC) were also employed to model SARS-CoV-2 infection (5, 6). However, the disadvantages include fetal-like properties that appear to be inherent to iPSC-derived organoids and complicated manipulations to generate these organoids. Respiratory organoids derived from primary human lung tissues and fetal lung bud tips were used to study SARS-CoV-2 respiratory infections (7–9). These organoids outperformed most in vitro models of cell lines, such as A549 and Calu-3, and primary epithelial cells.

We established the first organoid culture system of the human respiratory epithelium directly from primary lung tissues and nasal epithelial cells (10–14). These respiratory organoids were stably and consecutively expanded over half a year to one year. We induced differentiation in long-term expandable organoids and generated mature airway and alveolar organoids containing all major cell populations in the native airway and alveolar epithelium, respectively (10, 12, 13). Nasal organoids, which were derived from nasal epithelial cells of healthy donors with excellent efficiency, contained the four airway epithelial cell types, similar to airway organoids. Besides the noninvasive procedure to acquire nasal cells and superior efficiency in deriving organoid culture, nasal organoids differ from airway organoids in that the former retains the attributes of the upper airway epithelium. All the differentiated respiratory organoid models, including nasal, airway, and alveolar organoids, adequately simulate the multicellular composition of the native respiratory epithelium at different anatomical locations and phenocopy the related functionality, including mucociliary escalator in human airways, and type II alveolar epithelial cell secretion and recycling of surfactant protein as seen in the human alveolus (10, 12, 13, 15). These organoids also reproduced the expression profile of ACE2 and TMPRSS2, the essential host factors for SARS-CoV-2 infection, in the human upper and lower respiratory epithelium (16). Overall, the organoid culture system enabled us to reconstruct and expand the entire human respiratory epithelium in culture dishes with excellent efficiency and stability, providing an almost infinite amount of native human respiratory epithelial cells for routine experimentation. Notably, we demonstrated that mature airway organoids, especially nasal organoids, adequately recapitulated SARS-CoV-2 upper respiratory infection and higher transmissibility of Omicron subvariant B.1.1.529 (12, 13).

The surging case number of BA.5 worldwide was an indisputable manifestation of its enhanced transmissibility over sister subvariants and prior variants. Lewnard et al. demonstrated that BA.4/BA.5 infection cases were associated with reduced protection from COVID-19 vaccination and prior infection (17). Massively accumulating evidence showed reduced neutralization of emerging variants by prior immunity, including the newly emerging variants Omicron BA.1, BA.2, BA.4, and BA.5 (18–22), which was believed to drive the enhanced viral transmissibility in the community. Nonetheless, we suppose that immune evasion would not be the sole strategy for the virus to become more transmissible. Indeed, it was demonstrated that SARS-CoV-2 has evolved to gain replicative fitness. D614G was the first fitness-enhancing mutation arising within a few months of the COVID-19 pandemic (23). Variants carrying a spike L452R mutation exhibited increased infectivity in vitro and viral shedding in vivo, as well as decreased antibody neutralization (24). BA.4/5 spike gained a slightly higher affinity for the ACE2 receptor than that of an ancestral strain (20), carried the notable L452R mutation and demonstrated an enhanced fusogenic activity (22, 25). The phenotypic consequences of emerging mutations and variants were examined and demonstrated in cell-free and cell-based assays developed meticulously. Despite the artificial setting, these assays indeed analyzed and elucidated biological activities during virogenesis. To unravel the mechanism(s) leading to the increasing transmissibility, apart from the well-documented immune escape, we have to address a central issue: SARS-CoV-2 replicative capacity or intrinsic fitness in human respiratory cells, i.e., the ultimate outcome of proviral and antiviral alterations taking place in these cells. The replicative efficiency of newly emerging variants was assessed in iPSC-derived respiratory organoids, showing higher viral gene copy numbers in BA.5 infection than BA.2 infection (18, 22, 26). Yet, viral replication seemed less prominent in these cells; BA.5 only exhibited a modest or even marginal growth advantage over prior variants.

To enable efficient transmission, human RNA viruses evolve and constantly mutate with altered antigenicity to evade prior immunity and/or enhanced replicative fitness. In contrast to the massive documentation of immune escape, SARS-CoV-2 intrinsic fitness in human respiratory cells, the primary target of SARS-CoV-2, has remained incompletely understood, owing to the lack of a robust in vitro tool with easy accessibility and biological relevance to human respiratory cells. The respiratory organoids we have established and meticulously characterized provided an optimal in vitro correlate of the human respiratory epithelium to evaluate the replicative fitness of emerging respiratory viruses (10, 12, 13, 15). With these physiologically active respiratory epithelium models, we sought to elucidate whether SARS-CoV-2 has evolved to gain increased replicative fitness and whether BA.5 has become well adapted in human respiratory cells.

## Results

### The Highest Replicative Fitness of BA.5 in Airway and Nasal Organoids.

**Fig. 1.**
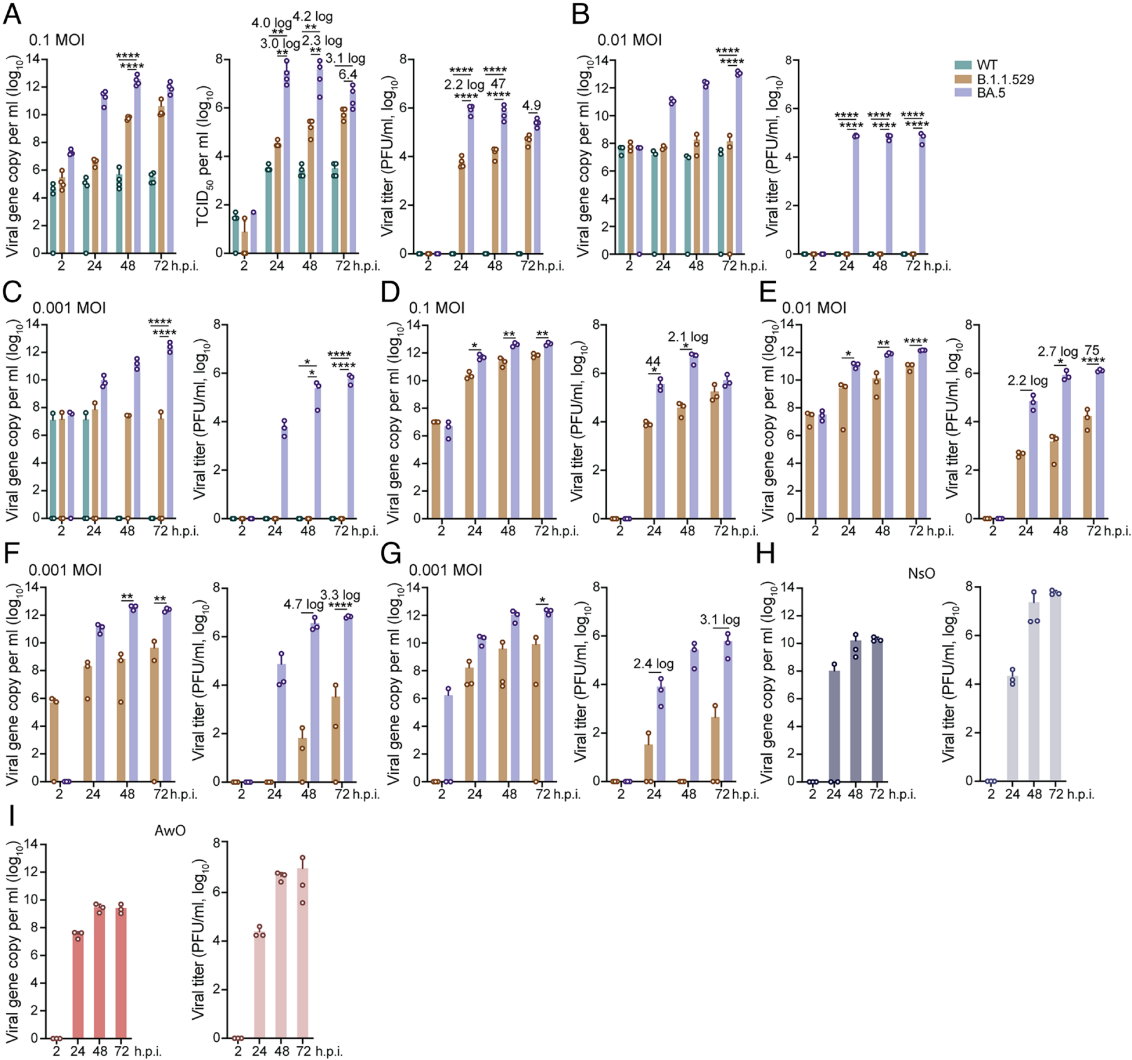
SARS-CoV-2 infection and replicative fitness in human airway and nasal organoids. (*A*–*C*) Airway organoids were inoculated with WT, B.1.1.529, and BA.5 at 0.1 multiplicity of infection (MOI) (*A*, n = 4), 0.01 MOI (*B*, n = 3), 0.001 MOI (*C*, n = 3). Culture media were harvested from infected airway organoids at the indicated hours postinoculation and applied to the detection of viral replication by the RT-PCR, TCID_50_, and plaque assay. Statistical significance was determined using two-way ANOVA with Tukey’s multiple comparisons test. (*D*–*G*) Nasal organoids were inoculated with B.1.1.529 and BA.5 at an MOI of 0.1 (*D*), 0.01 (*E*), and 0.001 (*F* and *G*). At the indicated hours postinoculation, culture media were harvested and applied to viral load detection by RT-qPCR and viral titration by the plaque assay. n = 3. Statistical significance was determined using a two-tailed Student’s *t* test. Nasal organoids (*H*) and airway organoids (*I*) were inoculated with a pandemic H1N1 virus at 0.001 MOI. Culture media were harvested from infected organoids at the designated time points for viral load detection and viral titration by the plaque assay. Data represent mean and SD from a representative experiment. n = 3. *P < 0.05, **P < 0.01, ***P < 0.001, ****P < 0.0001.

We first examined the replication kinetics of a clinical isolate of BA.5 in airway organoids in comparison with a B.1.1.529 isolate and an ancestral HKU-001a strain (wildtype, WT). We only used monolayers of the airway and nasal organoids (hereafter, airway and nasal organoids) seeded on transwell inserts for experimentations since these monolayers sustained more robust SARS-CoV-2 growth than their 3D counterparts (12, 13). We harvested cell-free culture media from infected airway organoids after 0.1 multiplicity of infection (MOI), and examined viral growth by detecting viral gene copy numbers and viral titration. B.1.1.529 replicated to a significantly higher titer than WT, consistent with our previous findings (12, 13). Notably, BA.5 showed the highest replicative capacity, around 2 ~ 3 log units higher than B.1.1.529, and more than 4 log units higher than WT as detected by the TCID_50_ assay (Fig. 1 *A*, *Left* and *Middle*). BA.5 reached a peak viral titer of over 7 log units/mL at 24 hours postinfection (h.p.i.), which was the highest viral titer we have ever detected in SARS-CoV-2-infected respiratory organoids.

We routinely propagated SARS-CoV-2 viruses in VeroE6-TMPRSS2 cells and also titrated the viruses in these cells using the plaque assay. However, titration of virions released from SARS-CoV-2-, especially WT-, infected organoids, was performed with the TCID_50_ assay since the virions released from infected airway organoids did not consistently form clear plaques in VeroE6-TMPRSS2 monolayers, although they indeed developed cytopathic effect (CPE) in the monolayers in the TCID_50_ assay. The high viral titer of BA.5 and more prominent CPE observed in the TCID_50_ assay prompted us to perform the plaque assay using the same culture medium samples as those for the TCID_50_ assay. We found virions released from B.1.1.529- and BA.5-inoculated airway organoids generated typical plaques in VeroE6-TMPRSS2 monolayers (*SI Appendix*, Fig. S1). However, no plaque was discernible after inoculating the culture media from WT-infected airway organoids. The plaque assay revealed a robust replication of BA.5, with a peak viral titer 2.2 log units higher than that of B.1.1.529 (Fig. 1 *A*, *Right*), similar to its growth advantage shown by the TCID_50_ assay.

We typically examined viral replication kinetics after inoculation at 0.1 MOI since the prior SARS-CoV-2 strains, especially WT, did not consistently replicate in airway organoids after inoculation at a lower MOI. As shown in Fig. 1*A*, the viral titer of BA.5 peaked at 24 h.p.i. and decreased at 72 h.p.i., suggesting BA.5 may be able to establish a productive infection with a lower MOI. Thus, we inoculated airway organoids with the three virus strains at 0.01 and 0.001 MOI. Similar to our prior observations, viral load detection and viral titration indicated that WT and B.1.1.529 barely replicated in airway organoids after low MOI inoculations (Fig. 1*B*). Yet, BA.5 robustly propagated as expected. After inoculation at 0.001 MOI, we observed a dramatic increase in viral titer of around 6 log units within the 3-d observation period (Fig. 1*C*). The remarkable growth advantage of BA.5 over B.1.1.529 and WT, and the requirement of high MOI for B.1.1.529 and WT replication were reproduced in the airway organoids derived from another donor, although B.1.1.529 and WT replicated modestly after low MOI infections (*SI Appendix*, Fig. S2).

We then assessed the replication kinetics of B.1.1.529 and BA.5 in nasal organoids, an organoid model of the human upper airway epithelium. After inoculation at 0.1 MOI, the viral titer of BA.5 in the culture medium was around 2 log units higher than that of B.1.1.529 (Fig. 1*D*). When nasal organoids were inoculated at 0.01 MOI, the growth advantage of BA.5 over B.1.1.529 was further augmented (Fig. 1*E*). Again, BA.5 robustly replicated after inoculation at 0.001 MOI, showing a significantly higher viral load and viral titer (4.7 log units higher) than B.1.1.529 at 48 h.p.i. (Fig. 1*F*). In the nasal organoids derived from another donor, BA.5 invariably replicated more actively than B.1.1.529 after inoculation at 0.001 MOI (Fig. 1*G*). We also examined the viral growth of clinical isolates of BA.4 and BA.2.12.1, together with BA.5 in the airway organoids from two different donors. After inoculation at 0.001 MOI, all three virus strains replicated to a high and comparable titer (*SI Appendix*, Fig. S3). We examined the replication kinetics of these two isolates, together with WT, B.1.1.529 and BA.5 in VeroE6-TMPRSS2 cells in parallel. In contrast, WT replicated more actively than all Omicron subvariants in VeroE6-TMPRSS2 cells (*SI Appendix*, Fig. S4).

To test whether BA.5 acquired a replicative fitness comparable to well-adapted human respiratory viruses, we infected the nasal organoids (Fig. 1*H*) and airway organoids (Fig. 1*I*) with a seasonal influenza A virus strain, a pandemic H1N1 virus (H1N1pdm), in parallel. We observed a significantly increased viral load and viral titer after infection at 0.001 MOI, as we demonstrated previously (10). Of note, BA.5 displayed comparable replication kinetics to H1N1pdm. Collectively, the results indicate that human airway and nasal organoids are more susceptible to BA.5 than the prior variants B.1.1.529 and WT; BA.5 acquired an adequate fitness in human airway and nasal organoids to a level comparable to a seasonal influenza virus strain.

### Higher Infectivity of Live BA.5 Virus and More Efficient Cell Entry of BA.5 Pseudovirus Particles.

**Fig. 2.**
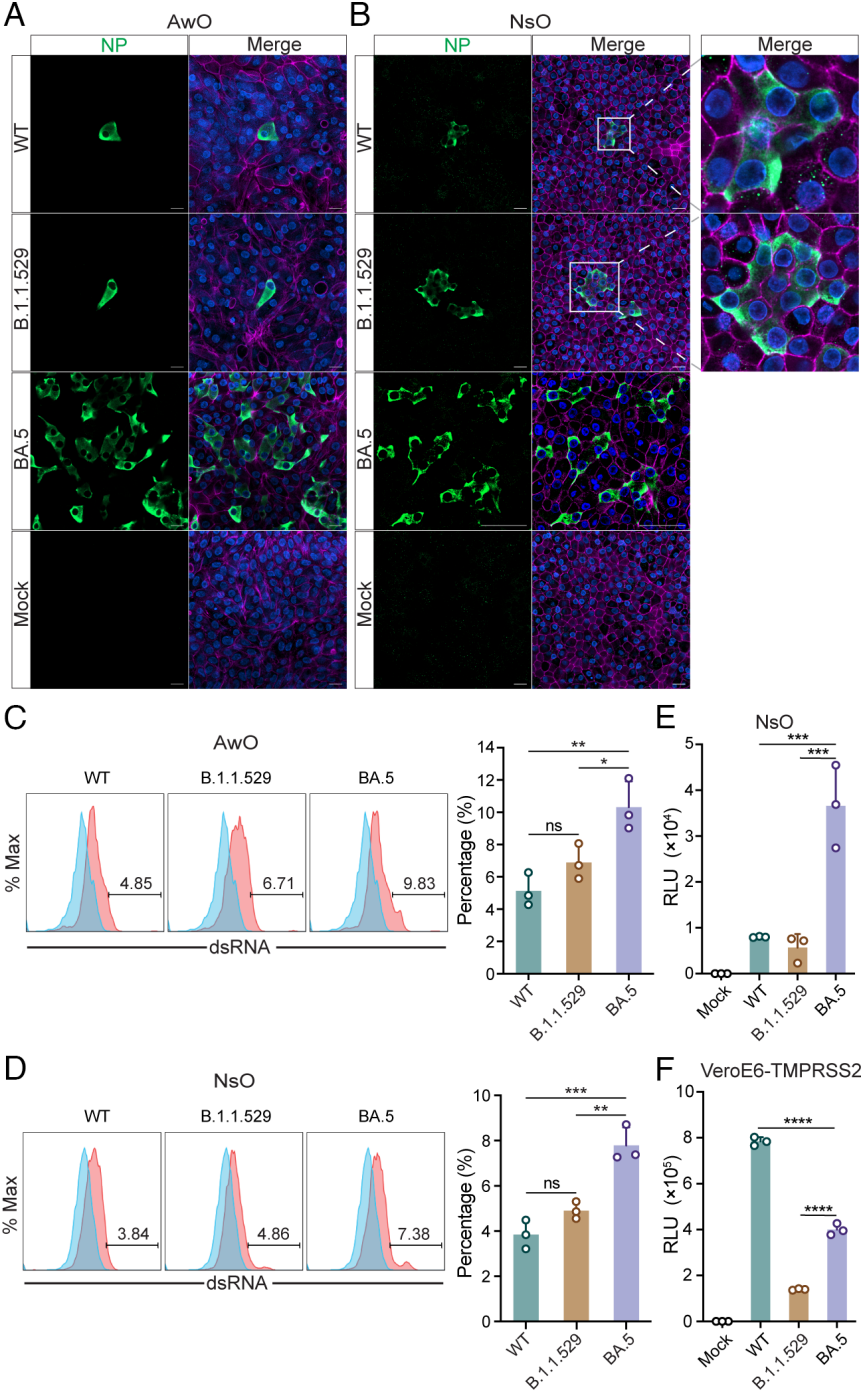
Higher infection rate and entry efficiency of BA.5 in human airway and nasal organoids. (*A* and *B*) Airway (*A*) and nasal organoids (*B*) were inoculated with SARS-CoV-2 WT, B.1.1.529, and BA.5 at 0.01 MOI. At 24 hours postinoculation (h.p.i.), the infected organoids were fixed and applied to immunostaining of SARS-CoV-2 viral NP (green). Nuclei and actin filaments were counterstained with DAPI (blue) and Phalloidin-647 (purple), respectively. (Scale bar, 20 µm.) (*C* and *D*) WT-, B.1.1.529-, and BA.5-infected airway (*C*) or nasal (*D*) organoids were dissociated after fixation, then applied to immunostaining and flow cytometry to detect the percentage of virus-infected cells. Representative histograms are shown on the *Left*. Data presented on the right represent mean and SD from a representative experiment. n = 3. (*E* and *F*) Nasal organoids and VeroE6-TMPRSS2 cells were infected with lentiviruses pseudotyped with the spike of WT, B.1.1.529, and BA.4/5. At 72 h.p.i., cell lysates of the infected nasal organoids (*E*), and VeroE6-TMPRSS2 cells (*F*) were applied to the luciferase assay. Data represent mean and SD from a representative experiment. n = 3. Statistical significance was determined using one-way ANOVA with Tukey’s multiple comparisons test. *P < 0.05, **P < 0.01, ***P < 0.001, ****P < 0.0001. ns, not significant.

To further characterize the infection in airway and nasal organoids, we inoculated the organoids with WT, B.1.1.529, and BA.5 at 0.01 MOI, and fixed them for immunostaining and confocal imaging at 24 h.p.i. We observed a more prominent infection of BA.5 than B.1.1.529 and WT (Fig. 2*A*). Viral NP+ cells in BA.5-infected airway organoids were remarkably more abundant than those in B.1.1.529- and WT-infected organoids. BA.5 invariably showed a more productive infection in nasal organoids than the other two strains (Fig. 2*B*). We also performed flow cytometry analysis to detect the percentage of virus-infected cells in the airway and nasal organoids 24 h after infection at 1 MOI. BA.5 showed a significantly higher infection rate than B.1.1.529 and WT in the airway (Fig. 2*C*) and nasal organoids (Fig. 2*D*).

To decipher the mechanism leading to the higher infectivity of BA.5, we constructed the lentiviruses pseudotyped with the spike of BA.4/5, B.1.1.529, and WT. We infected nasal organoids with the pseudoviruses and measured spike-mediated viral entry at 72 h.p.i. As shown in Fig. 2*E*, BA.4/5 pseudovirus displayed a significantly higher entry efficiency than B.1.1.529 and WT pseudovirus in nasal organoids. We also performed the same experiment in VeroE6-TMPRSS2 cells, which showed a distinct profile of infectivity (Fig. 2*F*). The pseudovirus of WT showed the highest infectivity, followed by that of BA.5 and B.1.1.529, which was agreeable to the result of viral replication in VeroE6-TMPRSS2 cells (shown in *SI Appendix*, Fig. S4). Collectively, live virus and pseudovirus infection experiments suggest that BA.5 infects airway and nasal organoids with higher efficiency than B.1.1.529 and WT.

### Syncytium Formation in BA.5-Infected Nasal and Airway Organoids Exclusively.

We noticed apparent syncytium formation in nasal and airway organoids infected by BA.5 during confocal imaging, which was absent and had never been observed in organoid infections of prior strains we have ever tested, including Delta containing the D614G mutation that was documented to induce more intensive syncytium formation in cell lines than ancestral strains. SARS-CoV-2 spike-mediated syncytium formation in VeroE6 cells or other cell lines has been widely used as assays to measure fusogenic activity (27, 28), which facilitates virus spread and immune evasion. Syncytial bodies positive for pneumocyte markers were identified in the autopsy lung tissues of deceased COVID-19 patients (29, 30), an in vivo manifestation of the fusogenic activity of the SARS-CoV-2 spike. However, an extensive literature search only showed sporadic reports of syncytium formation in the human airway or bronchial epithelial cells in vitro. In a recent publication, the authors used “syncytium-like” or “multinucleated cells reminiscent of syncytia” to describe the clusters formed on the apical side of SARS-CoV-2-infected primary bronchial epithelial cells (31). Here, we think it necessary to cautiously exclude the possibility that mucus secreted by goblet cells prompted an aggregation of the virus-infected cells undergoing CPE, rather than an authentic syncytium formation.

**Fig. 3.**
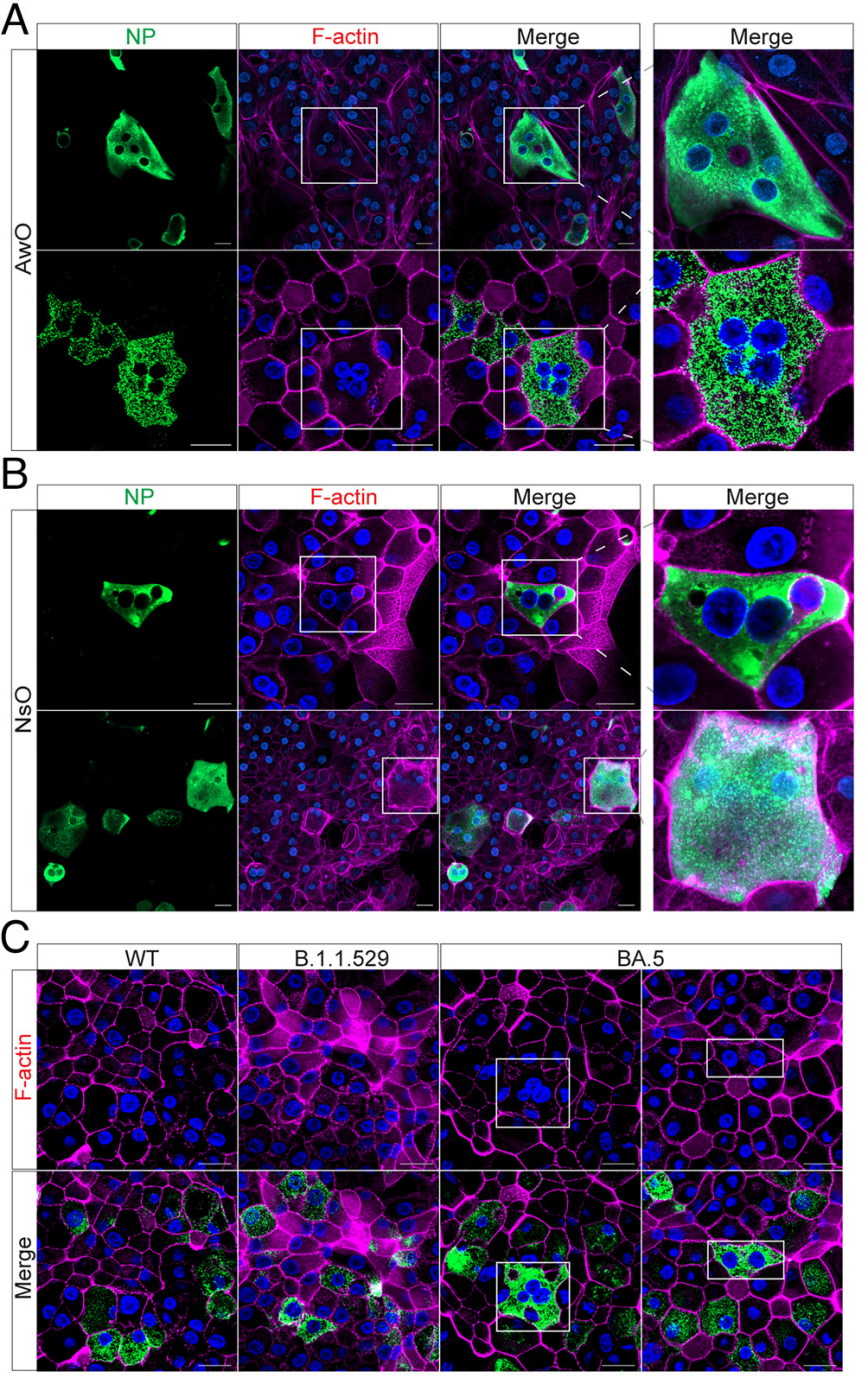
BA.5 infection and lentiviral overexpression of BA.5 spike-triggered syncytium formation in airway and nasal organoids. (*A* and *B*) Airway (*A*) and nasal organoids (*B*) were inoculated with SARS-CoV-2 BA.5 at 0.01 MOI. At 48 h.p.i., the infected organoids were fixed and applied to immunostaining of viral NP (green). Nuclei and actin filaments were counterstained with DAPI (blue) and Phalloidin-647 (purple), respectively. (Scale bar, 20 µm.) (*C*) Nasal organoids were transduced with lentiviruses overexpressing SARS-CoV-2 WT, B.1.1.529, and BA.4/5 spike. At 48 h posttransduction, the transduced organoids were fixed with 4% PFA and applied to immunostaining using an anti-FLAG antibody, followed by confocal imaging. Nuclei and actin filaments were counterstained with DAPI (blue) and Phalloidin-647 (purple), respectively. (Scale bar, 20 µm.)

In the present study, multinucleated syncytium was indiscernible in airway and nasal organoids infected with WT and B.1.1.529, consistent with our prior observations. Viral NP+ cells may cluster in the WT- and B.1.1.529-infected organoid monolayers, especially when a more active infection occurred in nasal organoids (WT and B.1.1.529 in Fig. 2*B*). However, individual infected cells within the cluster had a clear boundary delineated by cellular F-actin filaments, like a sheet of mosaic tiles. Notably, NP+ multinucleated syncytia with typical morphology were readily discernible in BA.5-infected monolayers of the airway organoid (Fig. 3*A*) and nasal organoids (Fig. 3*B*). Of note, F-actin filaments were dismantled or disappeared within the syncytia, indicating that BA.5 infection indeed triggered the formation of typical syncytia. BA.5-induced syncytia normally contained 2 to 5 nuclei in confocal images, large syncytia with over 10 nuclei were also discernible (*SI Appendix*, Fig. S5).

The presence of syncytial bodies in the live BA.5-infected airway and nasal organoids suggested that BA.5 spike possessed a substantially elevated fusogenic activity than the spike of prior variants, which was sufficiently potent to overcome the well-developed cellular junctions in these organoids and enabled syncytium formation. SARS-CoV-2 spike was able to induce syncytium in neighboring ACE2-expressing cells, and TMPRSS2 enhanced the syncytium formation (32). To verify the above findings, we overexpressed the spike protein of BA.4/5, B.1.1.529, and WT via lentiviral transduction to examine syncytium formation in the organoids that possess endogenous ACE2 and TMPRSS2 (12). Immunostaining showed that WT and B.1.1.529 spike were expressed in individual cells within the organoids (Fig. 3*C*). Similarly, no syncytial body was discernible. In contrast, BA.5 spike overexpression resulted in the formation of spike-positive clusters (Fig. 3*C*), within which cellular actin filaments were largely disrupted, very similar to the multinucleated syncytium in BA.5-infected organoids. The dismantled actin filaments in spike-positive syncytia were in stark contrast to the delicate actin filaments in neighboring spike-negative cells. Yet, the size of syncytia induced by exogenous overexpression of the BA.5 spike was generally smaller with fewer nuclei than those present in BA.5-infected organoids. Collectively, live virus infection and spike overexpression indicate that, among our tested virus strains, only BA.5 spike triggers the formation of multinucleated syncytia in nasal and airway organoids.

### WT, B.1.1.529, and BA.5 Preferential Usage of TMPRSS2 for Cellular Entry and Viral Growth.

**Fig. 4.**
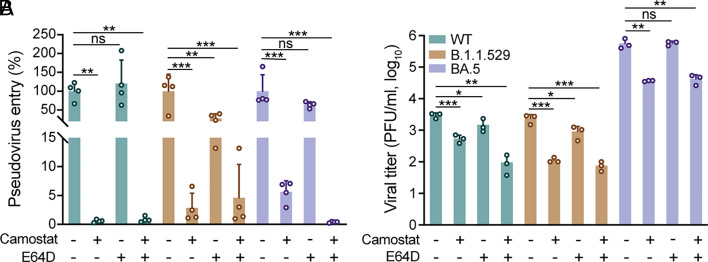
Preferential utilization of TMPRSS2 for cellular entry and viral replication by three strains of viruses. (*A*) Nasal organoids were inoculated with lentiviruses pseudotyped with the spike of WT, B.1.1.529, and BA.4/5 in the presence or absence of Camostat and E64D. At 72 h.p.i., cell lysates of infected nasal organoids were applied to the luciferase assay. n = 4. (*B*) Nasal organoids were inoculated with SARS-CoV-2 WT, B.1.1.529, and BA.5 at 0.1 MOI in the presence or absence of Camostat and E64D. At 24 h.p.i., culture media were harvested for viral titration by the plaque assay. n = 3. Data represent mean and SD from a representative experiment. Statistical significance was determined using one-way ANOVA with Tukey’s multiple comparisons test. *P < 0.05, **P < 0.01, ***P < 0.001, ****P < 0.0001. ns, not significant.

SARS-CoV-2 spike protein is cleaved into S1 and S2 subunits by ubiquitous endopeptidase furin in virus-producing cells. The S1 subunit binding to ACE2 primes a secondary cleavage at the S2′ site by serine protease TMPRSS2 at the surface plasma membrane or cysteine protease Cathepsin L in the endosomal compartment. TMPRSS2 or Cathepsin L cleavage of the S2′ site exposes the concealed fusion peptide, and triggers the fusion of viral and cellular membrane, followed by viral genome release to initiate replication. In Calu3 cells and human airway organoids, TMPRSS2-mediated cell entry represented the major route for SARS-CoV-2 infection (8, 33), whereas entry into TMPRSS2-deficient cell lines (e.g., Vero, HEK293T and A549) utilized the endosomal pathway mediated by Cathepsin L/B (34). To define the role of TMPRSS2 and Cathepsin L for viral entry, we measured WT, B.1.1.529, and BA.5 spike-driven pseudovirus entry into nasal organoids in the presence or absence of Camostat (a TMPRSS2 inhibitor) and E64D (a cathepsin L/B inhibitor). WT spike-driven entry was abolished by Camostat, but not E64D, indicating its dependence on TMPRSS2 to infect nasal organoids (Fig. 4*A*). B.1.1.529 and BA.5 spike also utilized TMPRSS2 for cellular entry, albeit to a slightly less extent than WT. E64D treatment decreased the entry of B.1.1.529 and BA.5 pseudoviruses, yet much less effectively than Camostat. Double treatment with Camostat and E64D abolished WT and BA.5 pseudovirus infectivity. Thus, TMPRSS2 appeared to be the dominant protease mediating cellular entry of all three variants into respiratory epithelial cells, and BA.5 and B.1.1.529 viruses utilized Cathepsin L to a less extent.

To elucidate the role of TMPRSS2 and Cathepsin L for viral replication, we measured viral titers of WT-, B.1.1.529-, and BA.5-infected nasal organoids with or without Camostat and E64D. Camostat treatment significantly reduced the viral titer of all three variants (Fig. 4*B*), whereas E64D treatment led to a slight viral reduction in WT and B.1.1.529. Double treatment with Camostat and E64D exaggerated the inhibition effect in WT and B.1.1.529, while it had no additive effect on BA.5. Thus, all three virus strains preferentially utilized TMPRSS2 for cellular entry and viral replication.

### Dampened Innate Immune Response upon SARS-CoV-2 Infection.

**Fig. 5.**
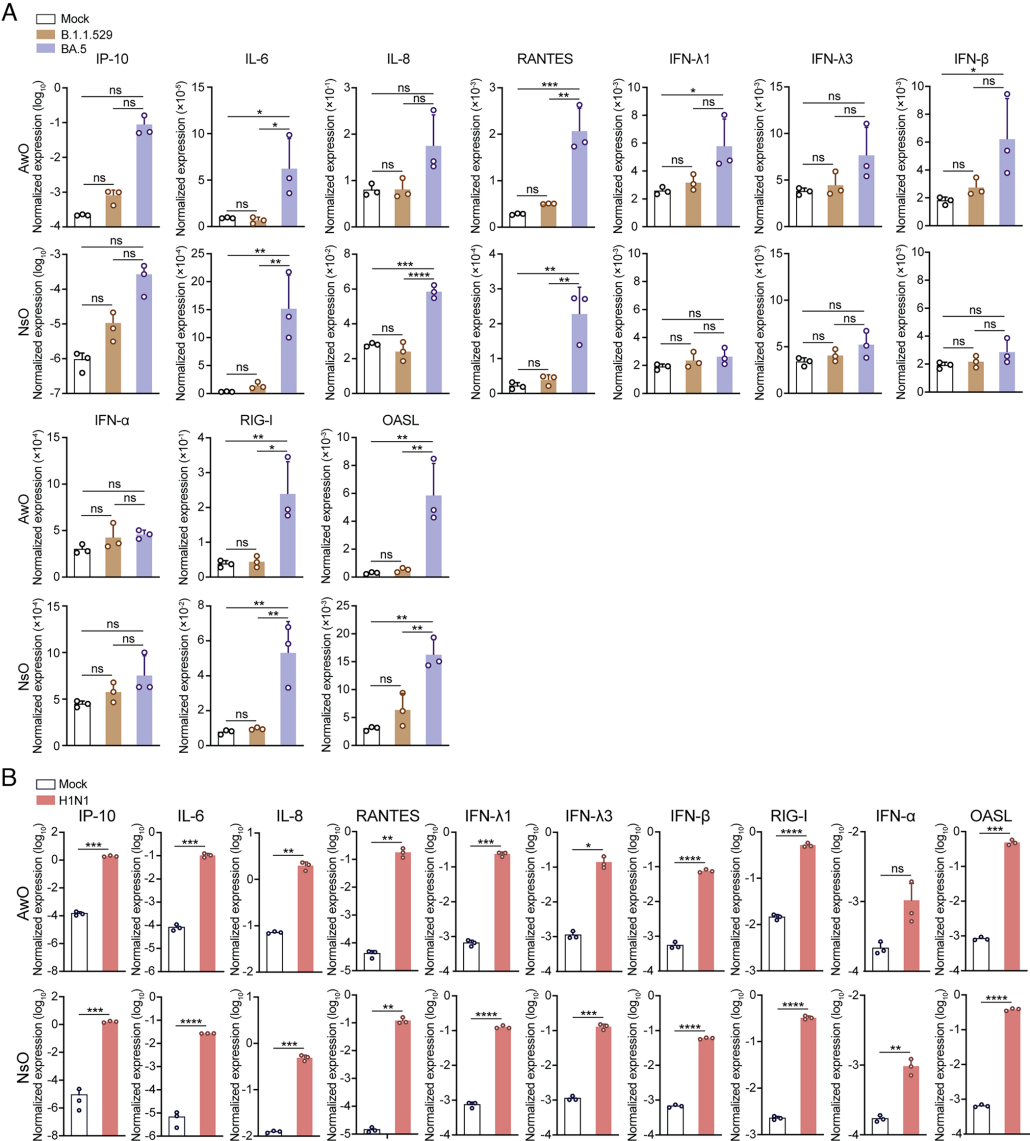
Dampened innate immune response in SARS-CoV-2 infected human airway and nasal organoids. (*A*) At 48 h.p.i. of B.1.1.529, BA.5 (0.1 MOI) in the airway or nasal organoids, cell lysates were harvested to examine GAPDH normalized expression levels of IFNs, ISGs, and proinflammatory cytokines in the virus-infected and mock-infected organoids with the RT-qPCR assay. Data represent mean and SD from a representative experiment. n = 3. Statistical significance was determined using one-way ANOVA with Tukey’s multiple comparisons test. *P < 0.05, **P < 0.01, ***P < 0.001, ****P < 0.0001. ns, not significant. (*B*) At 48 h.p.i. of H1N1pdm (0.1 MOI) in the airway or nasal organoids, cell lysates were harvested to examine GAPDH-normalized expression levels of IFNs, ISGs, and proinflammatory cytokines in the virus-infected and mock-infected organoids with the RT-qPCR assay. Data represent the mean and SD of a representative experiment in one organoid line. Data represent mean and SD from a representative experiment. n = 3. Statistical significance was determined using the two-tailed Student’s *t* test. *P < 0.05, **P < 0.01, ***P < 0.001, ****P < 0.0001. ns, not significant.

We further examined innate immune response in airway and nasal organoids after infection. After inoculation of B.1.1.529, BA.5 at 0.1 MOI, we harvested the organoids to detect the induction of IFNs, IFN-stimulated genes (ISGs) and proinflammatory cytokines in the infected organoids at 48 h.p.i. Relative to mock-infected organoids, we observed a dramatic IP-10 induction in BA.5-infected airway (403-fold) and nasal organoids (282-fold), respectively, with a higher magnitude than that in B.1.1.529-infected organoids (Fig. 5*A*). IL-6 underwent a significant induction of around 6.7- and 46.6- fold in BA.5-infected airway and nasal organoid, respectively. RANTES and IL-8 showed a modest induction profile in both organoids after BA.5 infection. IFNλs (IFNλ1 and IFNλ3), the major antiviral player in mucosal immunity (35, 36), were modestly induced by several folds in the BA.5-infected airway organoid and barely induced in B.1.1.529 infection. IFNβ showed a similar induction profile in BA.5-infected airway organoid, while IFNα was barely up-regulated. Yet, these IFNs were not induced in nasal organoids, even in BA.5 infection. Although IFNs were induced differentially in the airway and nasal organoids, ISGs such as 2′-5′-Oligoadenylate Synthetase Like (OASL) and RIG-I, were consistently up-regulated in BA.5-infected airway and nasal organoids, probably owing to the stimulation of some ISGs independent of IFN response (37).

Given the similar replicative kinetics of BA.5 and H1N1pdm, we detected these antiviral and proinflammatory genes in airway and nasal organoids after H1N1pdm infection at the same MOI. In stark contrast to the modest IFN induction in SARS-CoV-2 infection, IFNβ, especially IFNλ1 and IFNλ3 underwent a dramatic upregulation of over 2 log units in airway and nasal organoids (Fig. 5*B*), recapitulating the role of type III IFNs to be a major player in mucosal immunity. Accordingly, OASL and RIG-I were dramatically up-regulated 2 to 3 log units. IP-10, IL-6, IL-8, and RANTES also exhibited a prominent upregulation of over 1 to 3 log units. Overall, influenza virus infection in the organoids elicited an intensive upregulation of antiviral and proinflammatory genes. In contrast, SARS-CoV-2 infection in these organoids with similar replication efficiency exhibited a dysregulated pattern, including the strong induction of proinflammatory chemokines (IP-10) and dampened antiviral response of IFNs and ISGs.

### Attenuated Replication of BA.5 in Alveolar Organoids.

**Fig. 6.**
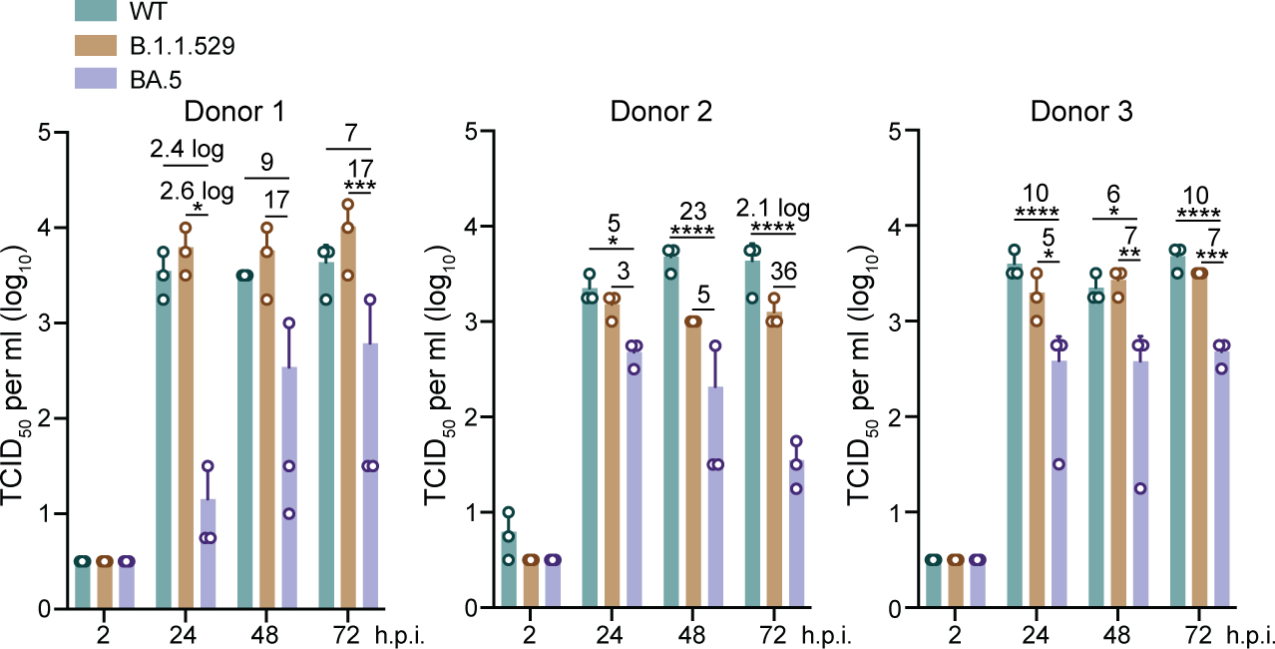
Attenuated BA.5 replication in human alveolar organoids. Alveolar organoids from three donors were inoculated with SARS-CoV-2 WT, B.1.1.529, and BA.5 at 0.1 MOI. Culture media were harvested from infected organoids at the designated time points for viral titration by the TCID_50_ assay. Data represent mean and SD from a representative experiment in one line of organoids. n = 3. Statistical significance was determined using two-way ANOVA with Tukey’s multiple comparisons test. *P < 0.05, **P < 0.01, ***P < 0.001, ****P < 0.0001.

We examined the replication kinetics of WT, B.1.1.529, and BA.5 in alveolar organoids that faithfully recapitulate the cellular composition, morphological features and functionality of the native alveolar epithelium (12). We inoculated alveolar organoids from three donors with WT, B.1.1.529, and BA.5 at 0.1 MOI. Consistent with prior observations of ours and others (12, 16), all three viruses replicated modestly with a peak viral titer of around 4 log units/mL (Fig. 6), which reproduced the low susceptibility of human alveolar epithelial cells to SARS-CoV-2. Although vital titer in the culture media showed a slight variation among the organoids from the three donors, the viral titer of BA.5 was consistently and significantly lower than that in WT and B.1.1.529, suggesting the lower propensity of BA.5 to cause pneumonia, a severe complication of COVID-19.

## Discussion

In the past 2 y, we have witnessed the rapid evolution of SARS-CoV-2. Variants of increasing transmissibility have emerged, which has accelerated since late 2021. Immune evasion, which was extensively reported (18–22), is an effective mechanism for SARS-CoV-2 to enhance its transmissibility, but not the sole strategy. Several studies have demonstrated altered virological properties of SARS-CoV-2 newly emerging variants (20, 22). Yet, the replicative advantage of BA.5 seemed ambiguous and less convincing. A critical issue remained unresolved, whether SARS-CoV-2 has evolved to acquire higher fitness gradually, leading to escalating transmissibility. The major hurdle is the lack of robust and biologically relevant in vitro models, a correlate of the human respiratory epithelium, to elucidate viral infection and replication in human respiratory cells. The respiratory organoids we established provide a unique model system to address the critical question.

We demonstrated that BA.5 displayed a remarkably increased fitness than an ancestral strain HKU-001a (WT) and the Omicron subvariant B.1.1.529 in the human airway and nasal organoids (Fig. 1 and *SI Appendix*, Figs. S1–S3). The major player leading to enhanced fitness, we believe, is the exceptional ability of BA.5 spike to trigger syncytium formation in the airway and nasal organoids. Spike-mediated syncytium formation was well documented in cell lines and autopsy lung tissues of COVID-19 patients (29, 30); whereas syncytium formation in primary bronchial epithelial cells was controversial. We found that BA.5 infection triggered the formation of multinucleated syncytia in airway and nasal organoids (Fig. 3 and *SI Appendix*, Fig. S5), which was absent in our experimentations of prior strains we have tested. The human airway epithelium, especially the upper respiratory nasal epithelium, is the frontline of defense against the external environment, including invading viruses. It is conceivable that potent cell junctions are required to establish a strong and intact epithelial barrier in the respiratory mucosa against external stimuli, which was adequately revealed by the high transepithelial electrical resistance in monolayers of airway and nasal organoid (12, 13). Among Omicron subvariants, BA.5 carried an L452R mutation with multiple provial properties, one of which was an increased fusogenicity (25). Kimura et al. also described the higher fusogenic activity of BA.4/5 spike than the spike of other variants in a cell-based fusogenic assay (22). Thus, the heightened fusogenic capacity of BA.4/5 spike may overcome the well-developed cell junctions in the airway and nasal organoids and enable syncytium formation. We also verified the syncytium formation of BA.4/5 spike in the airway organoids via lentivirus overexpression of spike protein. The unique ability of BA.5 to form syncytia in airway and nasal organoids represents a “one stone two birds” strategy to achieve enhanced replicative fitness and immune escape simultaneously. In addition, the higher entry efficiency mediated by BA.5 spike, as illustrated in the pseudovirus infectivity assay and live virus infection experiments (Fig. 2), also contributed to its enhanced replicative fitness.

Notably, BA.5 achieved comparable replicative fitness to a seasonal influenza virus H1N1pdm (Fig. 1 *H* and *I*), suggesting that BA.5 has become well adapted in the human airway epithelial cells. Despite their comparable fitness in the airway and nasal organoids, the innate immune response triggered by BA.5 and H1N1pdm showed a strikingly different magnitude. Except for IP-10, all the antiviral genes and proinflammatory mediators were exceedingly higher in H1N1pdm infection than BA.5 infection (Fig. 5). The dampened proinflammatory response, which was previously reported in primary nasal epithelial cells and respiratory cells of COVID-19 patients (38, 39), may account for the high proportion of asymptomatic COVID-19 cases (40), especially in the presence of extensive herd immunity. The major antiviral cytokines, especially type III IFN, were modestly or barely induced in the B.1.1.529- and BA.5-infected organoids (Fig. 5*A*), in line with the strong IFN antagonism of SARS-CoV-2 as reported exclusively (41). The blunted antiviral response may further enhance SARS-CoV-2 propagation in respiratory epithelial cells. Overall, our studies in the airway and nasal organoids unraveled the mechanisms leading to the elevated fitness of BA.5, i.e., higher entry efficiency, syncytium formation, and blunted antiviral response. Fortunately, even after a high MOI (0.1) inoculation, we observed a modestly increased viral titer in alveolar organoids, indicating the lower susceptibility of alveolar epithelial cells to SARS-CoV-2. Moreover, BA.5 replication in alveolar organoids was significantly lower than that of WT and B.1.1.529 (Fig. 6), which recapitulated BA.5′ attenuated pathogenicity and the lower risk of developing pneumonia in COVID-19 patients.

Consistent with earlier observations in the airway organoids (8), BA.5, WT, and B.1.1.529, preferentially utilized TMPRSS2 for cellular entry and viral growth, and Omicron variants B.1.1.529 and BA.5 also used Cathepsin for endothermal entry to a less extent (Fig. 4), though more detailed characterizations, such as detection of IC_50_s of Camostat and E64D, for each virus strains are warranted to validate the results. The preferential usage of TMPRSS2 provided in vitro evidence for the efficacy of Camostat mesylate reported in a phase II clinical trial (42). Yet, our studies in nasal and airway organoids appeared to obtain disparate results from those of immortalized cell lines (43, 44), in which it was demonstrated that Omicron variants preferred the TMPRSS2-independent endothermal entry route. Here, we ascribed the disparate results to the fundamental distinction between our organoids and immortalized cell lines. In this study, we characterized the replicative fitness and infectivity of different variants via live virus infection, pseudovirus infection, and lentivirus transduction in physiologically relevant respiratory organoids. We also performed some experiments in parallel, e.g., replication kinetics and pseudovirus infection, in VeroE6-TMPRSS2 cells (*SI Appendix*, Fig. S4 and Fig. 2*F*). Similar to the discrepant results of entry routes in cell lines and our respiratory organoids, the side-by-side comparison showed strikingly different results. During the establishment of cell lines, cells isolated from native tissues undergo an array of alterations in order to adapt and grow onto plastic culture plates (45–47). Thus, the resultant cell lines represent the survivorship of adapted and altered cells, which are invariably homogeneous and remarkably distinct from in vivo cells in terms of cellular identity and functionality. The discrepant observations in organoids and cell lines underscored the strength of physiologically active respiratory organoids for modeling and studying SARS-CoV-2 and COVID-19 disease.

Host susceptibility to a respiratory virus is the outcome of an arms race between host immunity and intrinsic viral fitness in human respiratory epithelial cells, the primary target of respiratory viruses. The control of infectious diseases should focus on prevention and blocking community transmission. Yet, high fitness of Omicron emerging subvariants comparable to a seasonal influenza virus and its potent immune escape, together with insufficient mucosal immunity induced by the current intramuscular COVID-19 vaccination, may pose a significant challenge to preventing SARS-CoV-2 transmission. As such, highly efficient intranasal vaccination to elicit potent mucosal immunity, development of antivirals against SARS-CoV-2 and therapeutic agents might represent effective strategies to combat COVID-19.

## Materials and Methods

### Establishment, Maintenance, and Differentiation of Respiratory Organoids.

Several lines of human lung organoids were established previously using lung tissues from patients who underwent surgical resection owing to various diseases with written consent, upon approval by the Institutional Review Board of the University of Hong Kong/Hospital Authority Hong Kong West Cluster (UW21-695) as described previously (12). The normal lung tissues adjacent to the diseased tissues were used to derive organoids. After 1 ~ 2 passages of 2 to 3 wk, fibroblasts and other nonepithelial components in the initial organoids disappeared gradually (12). Afterward, the lung organoids exclusively consisting of epithelial cells proliferated and were stably expanded in the expansion medium for over 1 y (10, 12).

Nasal organoids were established from nasal epithelial cells noninvasively procured from healthy donors with written consent, upon approval by the Institutional Review Board of the University of Hong Kong/Hospital Authority Hong Kong West Cluster (UW21-695) (12). Nasal organoids were consecutively passaged in the expansion medium every 2 to 3 wk with a ratio of 1:3 to 1:10 for up to 6 mo (13). The proximal differentiation protocol for generating monolayers of the airway and nasal organoids was described previously (13, 15).

### Virus Infection and Detection.

SARS-CoV-2 isolate HKU-001a (WT, GenBank accession number MT230904), Omicron subvariant (B.1.1.529; GenBank OM212473), BA.5 (GISAID accession no. EPI_ISL_13777658), BA.4 (GISAID accession no. EPI_ISL_13777657) and BA.2.12.1 (GISAID accession no. EPI_ISL_13777659) were plaque purified and then propagated in VeroE6/TMPRSS2 cells (JCRB1819) purchased from Japanese Collection of Research Bioresources (JCRB) Cell Bank and titrated with the plaque assay as we described previously (48). Monolayers of human airway and nasal organoid were washed twice with the basal medium consisting of Advanced DMEM/F-12 (Gibco), 1% HEPES, 1% GlutaMAX, and 1% Penicillin/Streptomycin, followed by virus inoculation with the indicated MOIs for 2 h and subsequent incubation in the basal medium at 37 °C (12). After washing with basal medium, the differentiated human alveolar organoids were inoculated with the indicated virus at 0.1 MOI for 2 h and subsequently incubated in the basal medium (12). To assess replication kinetics, we harvested cell-free culture media at the indicated hours after infection, followed by RNA extraction using the MiniBEST Viral RNA/DNA Extraction Kit (Takara) and detection of viral loads with the RT-qPCR assay targeting the viral RNA-dependent RNA polymerase (RdRp) gene using the QuantiNova Probe RT-PCR Kit (Qiagen, 208354), and viral titration by the TCID_50_ assay and/or plaque assay as described previously (10, 12). To examine the role of TMPRSS2 and Cathepsin L for viral growth, after pretreatment with 50 µM Camostat (Sigma-Aldrich) or 50 µM E64D (Sigma-Aldrich) or DMSO for 1 h, we inoculated the organoid and VeroE6-TMPRSS2 monolayers with viruses with 0.1 MOI, then resumed the treatment of original inhibitor(s). At 24 h.p.i., culture media were harvested for viral titration by the plaque assay. All experiments with live viruses were conducted in biosafety level 3 laboratories after approval by the Faculty of Medicine, The University of Hong Kong.

### Immunofluorescence Staining, Confocal Imaging and Flow Cytometry.

To identify virus-infected cells, we stained virus- or mock-infected organoid monolayers and performed confocal imaging as described previously (12, 13, 36). Briefly, the fixed organoid monolayers were permeabilized with 0.1% Triton X-100 for 10 min and blocked with protein block (Dako) for 1 h, followed by incubation with an in-house antibody against SARS-CoV-2 nucleoprotein (NP) and secondary antibodies as described previously (12, 13, 36). Nuclei and actin filaments were counterstained with DAPI (ThermoFisher) and Phalloidin-Atto 647 (Sigma-Aldrich), respectively. After staining, the organoids were whole-mounted on a glass slide with ProLong™ Glass Antifade Mountant (Invitrogen) (12, 13). Confocal images were acquired using a Carl Zeiss LSM 800 confocal microscope. Image processing was performed using the ZEN blue software.

To detect infection rate by flow cytometry, we dissociated organoids into single cells with 10 mM EDTA (Invitrogen) for 30 to 60 min at 37 °C, 24 h after an MOI of 1 infection, followed by fixation with 4% PFA and permeabilization with 0.1% Triton X-100, and then applied to immunostaining using an α-dsRNA antibody (Scicons, 10010500) and a secondary antibody as described previously (12, 13). The mock-infected organoids were used for gating. BD FACSCantoII Analyzer or LSR Fortessa was used for analysis. FlowJo software was used for data processing.

### Pseudovirus Infectivity Assay.

Pseudoviruses were prepared by cotransfecting 16 μg pNL4-3.RE.luciferase plasmid and 8 μg pcDNA3.1 vector expressing spike gene of the indicated variant into Lenti-X 293T cells (Takara Bio) seeded in a 6-well plate. We harvested the culture medium 48 h posttransfection. After centrifugation and filtration, the luciferase activities of pseudoviruses were quantified using a GloMax Explorer Fully Loaded Model (Promega). To measure pseudovirus entry, we inoculated the monolayers of organoids or VeroE6-TMPRSS2 cells with pseudoviruses of 4 × 10^6^ units with 10 μg/mL polybrene. At 72 h postinoculation, the infected monolayers were applied to the luciferase assay using the Bright-Glo Luciferase assay system (Promega, E2620). In protease inhibition experiments, after pretreatment with 50 µM Camostat (Sigma-Aldrich) or 50 µM E64D (Sigma-Aldrich) or double treatment, or DMSO, the monolayers were subjected to the initial treatment and luciferase assay 72 h after incubation.

### Lentiviral Overexpression of Spike Protein.

The FLAG-tagged spike genes of WT, B.1.1.529, and BA.5 were inserted into a pLenti vector (Origene, PS100069). Lenti-X 293T cells seeded in a 6-well plate were cotransfected with three plasmids, including 12 μg pLenti-spike, 6 μg pspax2, and 6 μg pmd2g, using Lipofectamine 3000 (ThermoFisher). The culture media containing lentivirus particles were harvested 48 h posttransfection. After concentration with Amicon Ultra-15 Centrifugal Filter Unit (Millipore, UFC910008), we titrated the lentiviruses using a Lenti-X GoStix Plus kit (Takara Bio, 631280). Nasal organoid monolayers were transduced with lentiviruses at 5 MOI with 10 μg/mL polybrene. The transduced nasal organoids were fixed with 4% PFA at 48 h posttransduction and applied to immunostaining using an anti-FLAG (Sigma, F7425), followed by confocal imaging as described above (36).

### Statistical Analysis.

Statistical analysis was conducted using GraphPad Prism 9.0. Either Student’s *t* test or ANOVA test was used to determine statistical significance as specified in Fig. legends. The number of replicates is indicated in Fig. legends. *P ≤ 0.05, **P ≤ 0.01, ***P ≤ 0.001, **** *P* ≤ 0.0001.

## Supplementary Material

Appendix 01 (PDF)

## Data Availability

All study data are included in the article and/or *SI Appendix*.
