# Supplementary material for: Human airway and nasal organoids reveal escalating replicative fitness of SARS-CoV-2 emerging variants
Source: Proc Natl Acad Sci U S A. 2023 Apr 17;120(17):e2300376120. doi: 10.1073/pnas.2300376120 (PMC10151566; doi:10.1073/pnas.2300376120)
Supplement: Supplementary file 1 — Appendix 01 (PDF) [file pnas.2300376120.sapp.pdf]

PNAS

Supporting Information for

Human airway and nasal organoids reveal escalating replicative fitness of SARS-CoV-2 emerging variants

Cun Li[a,1], Jingjing Huang[a,1], Yifei Yu[a,1], Zhixin Wan[a,1], Man Chun Chiu[a], Xiaojuan Liu[a], Shuxin Zhang[a], Jian-Piao Cai[a], Hin Chu[a,b,c], Gang Li[d], Jasper Fuk-Woo Chan[a,b,c,e], Kelvin Kai-Wang To[a,b,c,e], Zifeng Yang[f], Shibo Jiang[g,2], Kwok-Yung Yuen[a,b,c,e,2], Hans Clevers[h,i,2], Jie Zhou[a,b,c,2]

[1] These authors contributed equally.

[2] **Correspondence to:** Hans Clevers, Email: h.clevers@hubrecht.eu Pharma, Research and Early Development of F. Hoffmann-La Roche Ltd, CH-4070 Basel, Switzerland; Jie Zhou, Email: jiezhou@hku.hk and Kwok-Yung Yuen, Email: kyyuen@hku.hk Department of Microbiology, The University of Hong Kong, 102 Pokfulam Road, Hong Kong, China; and Shibo Jiang, shibojiang@fudan.edu.cn School of Basic Medical Sciences, Fudan University, Shanghai 200032, China.

This PDF file includes:

Figures S1 to S5

Fig. S1.

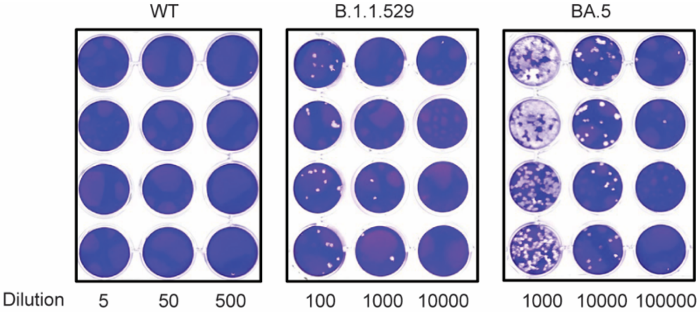

Fig. S1. Plaque assay in VeroE6-TMPRSS2 cells. Airway organoids were inoculated with SARS-CoV-2 WT, B.1.1.529, and BA.5 at 0.1 MOI. At the indicated h.p.i., culture media were harvested and applied to viral titration by plaque assay in VeroE6-TMPRSS2 cells. The representative photos of plaque formation in airway organoids infected by WT, B.1.1.529, and BA.5 are shown.

Fig. S2.

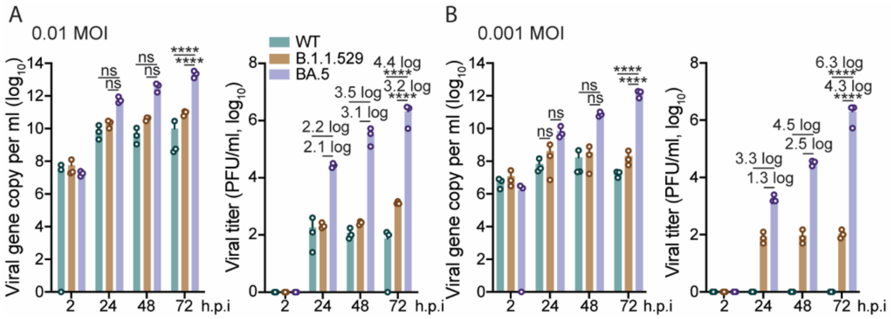

Fig. S2. Replication kinetics of SARS-CoV-2 WT, B.1.1.529, and BA.5 in airway organoids after low MOI infection. A, **B**, Airway organoids were inoculated with SARS-CoV-2 at 0.01 MOI (**A**), 0.001 MOI (**B**). Culture media were harvested from infected organoids at the designated time points for viral load detection and viral titration by plaque assay. n=3. Data represent mean and s.d. from a representative experiment. Statistical significance was determined using two-way ANOVA with Tukey's multiple comparisons test. *P < 0.05, **P < 0.01, ***P < 0.001, ****P < 0.001. ns, not significant.

Fig. S3.

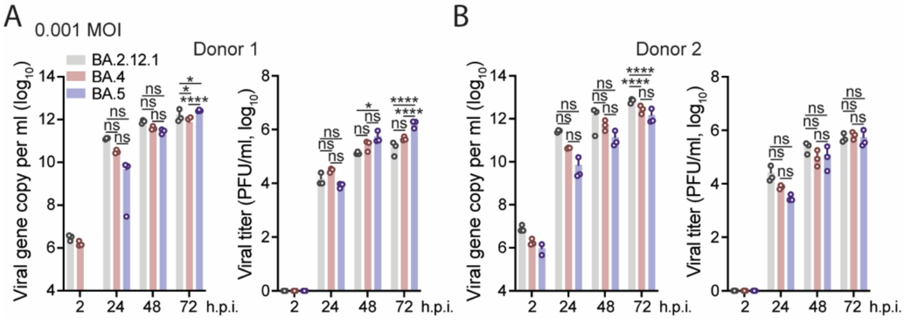

Fig. S3. Robust replication of SARS-CoV-2 BA.2.12.1, BA.4, and BA.5 in airway organoids. A, **B**, Airway organoids from two donors were inoculated with BA.2.12.1, BA.4, and BA.5 at 0.001 MOI. At the indicated h.p.i., culture media were harvested and applied to viral load detection by RT-qPCR and viral titration by plaque assay. Data represent mean and s.d. from a representative experiment in one line of organoids. n=3. Statistical significance was determined using two-way ANOVA with Tukey's multiple comparisons test. *P < 0.05, **P < 0.01, ***P < 0.001, ****P < 0.0001. ns, not significant.

Fig. S4.

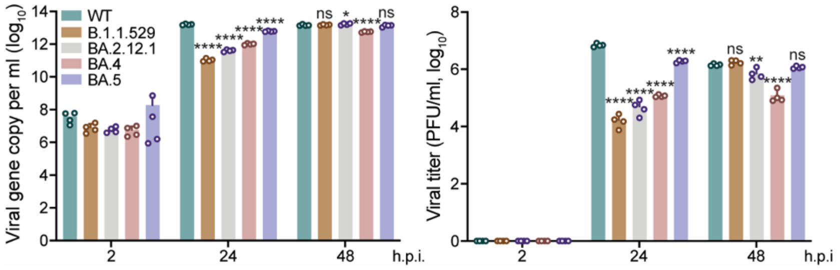

Fig. S4. Replication kinetics of SARS-CoV-2 variants in VeroE6-TMPRSS2 cells. VeroE6-TMPRSS2 cells were inoculated with WT, BA.1.1.529, BA.2.12.1, BA.4, and BA.5 at 0.01 MOI. At the indicated hours post-inoculation, culture media were harvested and applied to viral load detection by RT-qPCR and viral titration by plaque assay. Data represent mean and s.d. from are presentative experiment. n=3. Statistical significance was determined using two-way ANOVA with Tukey's multiple comparisons test. *P < 0.05, **P < 0.01, ***P < 0.001, ****P < 0.0001. ns, not significant.

Fig. S5.

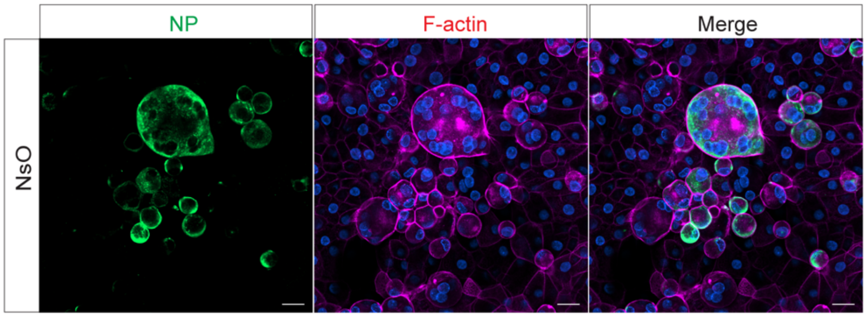

Fig. S5. BA.5 infection-triggered syncytium formation in nasal organoids. Nasal organoids were inoculated with SARS-CoV-2 BA.5 at 0.01 MOI. At 48 h.p.i., the infected organoids were fixed and applied to immunostaining of viral NP (green). Nuclei and actin filaments were counterstained with DAPI (blue) and Phalloidin-647 (purple), respectively. Scale bar, 20 µm.
